# Testing the buffering hypothesis: Breastfeeding problems, cessation, and social support in the UK

**DOI:** 10.1002/ajhb.23621

**Published:** 2021-05-30

**Authors:** Abigail E. Page, Emily H. Emmott, Sarah Myers

**Affiliations:** ^1^ Department of Population Health London School of Hygiene and Tropical Medicine London United Kingdom; ^2^ UCL Anthropology University College London London United Kingdom; ^3^ BirthRites Independent Max Planck Research Group Max Planck Institute for Evolutionary Anthropology Leipzig Germany

## Abstract

**Objectives:**

Physical breastfeeding problems can lead women to terminate breastfeeding earlier than planned. In high‐income countries such as the UK, breastfeeding problems have been attributed to the cultural and individual “inexperience” of breastfeeding, ultimately leading to lower breastfeeding rates. Yet, cross‐cultural evidence suggests breastfeeding problems still occur in contexts where breastfeeding is common, prolonged, and seen publicly. This suggests breastfeeding problems are not unusual and do not necessarily lead to breastfeeding cessation. As humans evolved to raise children cooperatively, what matters for breastfeeding continuation may be the availability of social support during the postnatal period. Here, we test the hypothesis that social support buffers mothers from the negative impact breastfeeding problems have on duration.

**Methods:**

We run Cox models on a sample of 565 UK mothers who completed a retrospective online survey about infant feeding and social support in 2017–2018.

**Results:**

Breastfeeding problems were important predictors of cessation; however, the direction of the effect was dependent on the problem type and type of support from a range of supporters. Helpful support for discomfort issues (blocked ducts, too much milk) was significantly associated with reduced hazards of cessation, as predicted. However, helpful support for reported milk insufficiency was assoicated with an *increased* hazard of cessation.

**Conclusions:**

Experiencing breastfeeding problems is the norm, but its impact may be mitigated via social support. Working from an interdisciplinary approach, our results highlight that a wide range of supporters who provide different types of support have potential to influence maternal breastfeeding experience.

## 1 INTRODUCTION

The middle and latter half of the 20th century saw a gradual replacement of breastfeeding with formula feeding in the UK (Tomori et al., 2016), a pattern mirrored in many high income contexts. In response to concerns regarding poorer health outcomes for infants fed on formula, breastfeeding promotion initiatives began growing in the 1970s (Stevens et al., 2009), culminating in breastfeeding becoming a global public health goal in the early 1990s, represented by UNICEF'S Baby Friendly Hospital Initiative which evolved into the Baby Friendly Initiative (BFI). In the UK, the BFI launched in 1994 providing guidance on 10 steps for hospitals to become “baby friendly,” including training healthcare staff to fully support breastfeeding, helping mothers initiate breastfeeding and work through challenges, and the promotion of breastfeeding (Fallon et al., 2019). Despite this public health attention and increased structural support for breastfeeding (Tomori et al., 2016), breastfeeding rates remain particularly low in the UK (Victora et al., 2016). While ~75% of UK mothers—similar to other European countries (Theurich et al., 2019)—initiate breastfeeding (Nuffield, 2021), only around 1% are exclusively breastfed to 6 months (McAndrew et al., 2012). Of particular interest, then, are the barriers to breastfeeding specifically in the UK. Here we focus on the experience of breastfeeding problems, a key predictor of breastfeeding cessation (Ingram et al., 2002), and assess whether social support acts as a buffer against them.

Breastfeeding problems are commonly reported in high‐income populations, with up to 80%–90% of mothers studied reporting problems (Bergmann et al., 2014; Lamontagne et al., 2008). Mothers have reported struggling with insufficient milk, sore and cracked nipples, painful breasts and poor latching (Ahluwalia et al., 2005; Binns & Scott, 2002; Ingram et al., 2002; Kirkland & Fein, 2003; Li et al., 2008). While the determinants of early breastfeeding cessation is multi‐faceted and complex (Thulier & Mercer, 2009), many women indicate physical breastfeeding problems as, for them, important reasons for terminating breastfeeding, particularly around perceived issues of breast milk insufficiency (Verronen, 1982). The experience of such problems in the early weeks of breastfeeding has also been indicated as key predictors of cessation in the UK and high‐income countries (HIC) generally (Ingram et al., 2002; McInnes & Chambers, 2008; Stuebe et al., 2014). Thus, breastfeeding problems may act as a barrier to continued breastfeeding, causing women who initiated breastfeeding to terminate earlier than planned. Yet, we know little about *how* various breastfeeding problems lead to early termination, especially since many women experience breastfeeding problems continue to breastfeed, and not all women see a “problem” as particularly problematic (Binns & Scott, 2002).

In the UK, breastfeeding problems are thought to be linked to women's lack of experience as breastfeeding is not commonly witnessed or discussed, and the “cultural value” of breastfeeding has been argued to be lost (Binns & Scott, 2002; Williamson et al., 2012). Women in the UK also report a lack of preparedness for the common problems of breastfeeding (Fallon et al., 2019; Hoddinott et al., 2012). In the UK, formula feeding has dominated in the recent past (Fomon, 2001), meaning that mothers are unable to draw on adequate breastfeeding knowledge and support from family and friends, and sometimes health care professionals (Fox et al., 2015; Hoddinott & Pill, 1999; Taylor et al., 2019). Therefore, a key factor amplifying the association between breastfeeding problems and breastfeeding termination in the UK may be a lack of support to help overcome the issues. Recent work by Scelza and Hinde (2019) with the Himba, a pre‐industrial pastoralist population from Namibia, demonstrated that while 63% of women reported some difficultly with breastfeeding—namely insufficient milk, pain and difficultly with latching—the Himba still had high breastfeeding rates, indicating the association between problems and cessation is not inevitable. In the Himba, breastfeeding is visible, on‐demand, and has occurred continuously over the generations. As a result, the community, and in particular grandmothers, have practical breastfeeding knowledge and experience which may “buffer” mothers from the adverse effects of breastfeeding challenges (Fox et al., 2015; Scelza & Hinde, 2019). While our focus is on the UK, such cross‐cultural and anthropological comparisons highlight diversity in the experience of social support and associations between breastfeeding problems and cessation (Van Esterik, 2018), providing valuable insights suggesting support as a potential mechanism moderating the experience and consequences of breastfeeding problems.

Here, we argue that social support may “buffer” against the negative impact of breastfeeding problems on breastfeeding duration in the UK. Increased access to breastfeeding knowledge as well as emotional and practical support may help mothers overcome the often unexpected challenges of breastfeeding (Hough et al., 2018), increasing women's ability to cope and deal with breastfeeding problems. Here, support is operationalized as a “resource transfer” from an individual supporter to the mother (Emmott et al., 2021; Myers et al., 2021). Such transfers or investments span informational, emotional, and practical domains, each predicted to buffer the mother in different ways from the potential negative influence of stressful events, such as breastfeeding problems (Cohen & Wills, 1985).

### 1.1 INFORMATIONAL SUPPORT

Informational support is the provision of advice, guidance, and information (House, 1981). In the face of such low levels of breastfeeding in the UK where less than 1% of infants are receiving any breast milk by 12 months (Victora et al., 2016), there is arguably a weakened culture of breastfeeding in the UK (Emmott et al., 2020b). This may mean potential supporters do not have effective breastfeeding information, limiting the “supportive” potential of informational support, as has been argued in a US context (Dennis & Faux, 1999). Medicalized interventions, in theory, often seek to fill this gap via the provision of informational support from health care professionals (HCP). Such support from HCP focuses on ensuring mothers have a good understanding of the physiology of lactation, are informed of the “right” breastfeeding technique to achieve optimal latch, as well as how to deal with common breastfeeding problems (Bergmann et al., 2014; Binns & Scott, 2002). As many first‐time mothers are unaware of how to breastfeed (Mcfadden et al., 2017) and mothers actively request breastfeeding tips (Graffy & Taylor, 2005), such support is beneficial (Mcfadden et al., 2017). However, a recent review of the UK BFI on breastfeeding outcomes found little evidence of sustained positive impact of the initiative on breastfeeding duration (Fallon et al., 2019). This work is in keeping with other systematic reviews which find interventions in the UK context to have limited or no impact on breastfeeding (Hoddinott et al., 2011; Jolly et al., 2012). Advice about “how to breastfeed” can vary in quality, may not address a mother's concerns or help with the problem. Furthermore, information given can be contradictory between sources and come morally loaded (Garner et al., 2016; Ingram et al., 2002; Lamontagne et al., 2008; McInnes & Chambers, 2008; Sriraman & Kellams, 2016). Therefore, while policy may emphasize informational breastfeeding support, in reality this support may not help mothers overcome difficulties (Rudzik, 2015; Tomori et al., 2018). Thus, it is important to separate out *helpful* and *unhelpful* support, as mothers may receive support which does not meet their needs or expectations.

### 1.2 EMOTIONAL SUPPORT

Emotional support is the conveyance of trust, empathy and concern, strengthening the individual's ability to cope (House, 1981). From an anthropological perspective, the importance of social support from a wide network is not surprising, given that humans have evolved a childrearing system which is reliant on the extensive help, support and guidance from a diverse range of individuals (Emmott & Page, 2019; Helfrecht et al., 2020; Hrdy, 2009; Page et al., 2019; Sear & Mace, 2008). Therefore, sharing experiences, receiving reassurance and emotional support from partners, family members and friends is likely important for mother and child outcomes, especially in the vulnerable perinatal period (Scelza & Hinde, 2019). Consequently, one limitation of public health approaches is a focus on maternal behavioral change facilitated by a narrow pool of professional supporters (Emmott & Mace, 2015; Hoddinott et al., 2011). Such approaches overlook mothers' wider social networks which provide emotional, as well as informational and practical support (Alianmoghaddam et al., 2019; Emmott et al., 2020a). Evidence suggests that maternal self‐efficacy is positively influenced by perception of social support from a diverse range of individuals (Ekström et al., 2003; Meedya et al., 2010; Wolfberg et al., 2004). This is particularly important for breastfeeding since low maternal confidence and reduced self‐efficacy may increase the perception of insufficient milk supply (given that only 5% of women face physiologically insufficient milk supply) (Hector & King, 2005; Kirkland & Fein, 2003; McCarter‐Spaulding & Kearney, 2001; Meedya et al., 2010). Milk release may also be impacted by emotional support, as emotional distress disrupts the release of oxytocin and prolactin inhibiting the milk ejection reflex (Dewey, 2001; Mohd Shukri et al., 2018), potentially leading to perception of low milk supply. Subsequent supplementation, to address perceived insufficiency, may then down‐regulate milk synthesis. Timely support to resolve concerns regarding supply, or other sources of distress, can prevent this chain of events. Further, receiving emotional support also increases a mother's capacity to cope with sore and cracked nipples and painful breasts (Ahluwalia et al., 2005; Binns & Scott, 2002; Ingram et al., 2002; Kirkland & Fein, 2003; Lamontagne et al., 2008; Li et al., 2008).

### 1.3 PRACTICAL SUPPORT

Practical support (also referred to as instrumental support) is the offer of physical help with tasks and changing the environment (House, 1981). While public health perspectives have primarily explored the importance of informational and emotional support, evolutionary anthropological perspectives have focused on the role of practical support (Scelza & Hinde, 2019). When individuals receive practical support through household tasks, resource provisioning, help with childcare, or allofeeding (where individuals other than the mother feed the infant), constraints on mothers are somewhat released (Emmott & Page, 2019; Kramer & Veile, 2018; Meehan et al., 2013; Page et al., 2021; Tully & Ball, 2018). In the context of breastfeeding problems, practical support may afford mothers the time, energy and space to focus on breastfeeding and work through the problems they are experiencing. Previous studies have documented a negative relationship between allofeeding and breastfeeding duration (Emmott et al., 2020b; Emmott & Mace, 2015; Rempel et al., 2017). However, mothers experiencing breastfeeding problems may benefit from following a mixed feeding strategy (e.g., combining breast and bottle), allowing sore nipples to heal, for instance, and may thus find allofeeding helpful, promoting continued breastfeeding. As with informational support, helpfulness is likely key to whether practical support facilitates overcoming or persevering with breastfeeding problems. While a mother may objectively receive practical support, if it does not assist with the particular issues with which she is dealing it is unlikely to alter her circumstance and, therefore, be considered helpful. It is crucial to take into account all aspects of support—including its helpfulness to the mother, type (informational, emotional and practical) and source—when unpacking relationship between problems and breastfeeding cessation.

## 2 RESEARCH QUESTION, HYPOTHESES, AND PREDICTIONS

We explore the multidimensional nature of social support from different supporters and its impact on breastfeeding problems and duration in the UK, bringing evolutionary anthropological perspectives focusing on practical support together with a public health emphasis on informational and emotional support. While the determinants of breastfeeding cessation are complex, including macro‐ and community‐level predictors, here we focus on the role of individual‐level experiences, namely breastfeeding problems and receipt of support. Specifically, we ask, does social support buffer mothers from the negative impact of breastfeeding problems on breastfeeding duration? We hypothesize that (H1) breastfeeding problems will increase the likelihood of breastfeeding cessation, and test the prediction that: all problems will be associated with increased likelihood of cessation (1a), except *too much breast milk* which we predict will be associated with a reduced likelihood of cessation (1b) (Ingram et al., 2002). Secondly, we hypothesize that (H2) social support, *if helpful*, will disrupt the negative relationship between breastfeeding problems and duration. We test the prediction that women who have breastfeeding problems who have ‘helpful’ levels of support will be less likely to stop breastfeeding, while those who have ‘unhelpful’ support will be more likely to stop breastfeeding (2a). Finally, as we are interested in the differential role of informational, emotional and practical support, we hypothesize that (H3) the size of the buffering effect will be dependent on the type of support; the role of informational support may be limited if it does not solve the problems mothers were facing. Specifically, we test the prediction that informational support will have less of a moderating effect on the breastfeeding problem‐cessation relationship compared with emotional and practical support (3a).

## 3 METHODOLOGY

### 3.1 Data and variables

We use data from a retrospective online survey developed as part of a wider project on social support and maternal experience (https://osf.io/7kb5q/) and hosted on Opinio in 2017–2018 (Table S1). Eligibility conditions included currently residing within the UK and giving birth to their last child within the UK in the last 24 months. Of 738 eligible individuals, 625 (84.7%) provided a measure of breastfeeding initiation and/or duration. Forty‐five women reported never initiating breastfeeding and 14 women skipped the social support questions and, thus, were removed. One individual did not record a stopping date and was listwise deleted from the analysis leaving a final sample size of 565.

#### 3.1.1 Breastfeeding duration

If women reported having ever breastfed their youngest child, they were asked if they were still giving *any* breast milk (either exclusively, supplemented with formula, or solid foods) and if they had stopped, how long they breastfed for (in days, weeks, or months). Mothers who reported breastfeeding for longer than 3 months or were still breastfeeding and the child was older than 3 months were right censored (i.e., individuals who breastfeed for longer where given duration lengths of 12.999 weeks) as previous research has demonstrated that breastfeeding problems are most severe and negatively correlated with duration within the first months following childbirth (Ahluwalia et al., 2005; Binns & Scott, 2002; Ingram et al., 2002).

#### 3.1.2 Breastfeeding problems

To assess physical breastfeeding problems, mothers were asked which of the following issues they experienced: sore/cracked/blistered nipples; not enough breast milk; too much breast milk/breast engorgement; mastitis; blocked milk ducts; thrush infection in the breast; problems with latching; and tongue‐tie (a shortening of the connection between the mouth and tongue, restricted tongue movement). Mothers could report multiple problems, meaning problems could co‐occur. Mothers could also select “other” and self‐report other problems as free text. No one raised further physical breastfeeding problems, suggesting our measure captures the key types of physical breastfeeding problems experienced by mothers in our sample. This created eight binary variables (1 = suffered from the problem).

#### 3.1.3 Support variables

Practical and informational support was captured in two questions to ensure we had an objective measure of support and how helpful this support was to participants. Firstly, we asked participants to report who they received specific types of support from (Table S1 for a breakdown), stating in the question “*regardless of whether they found it helpful*.” We then asked participants to rate how helpful they found this support, independent from the amount and/or type of support received. This ensures our measures do not conflate intention with actual helpfulness to the mother.

Participants were asked about the practical support they received during the first few weeks after giving birth from: their partner, mother (maternal grandmother), father (maternal grandfather), sibling(s), friends and the infant's paternal grandmother and grandfather. Participants were then asked to rate how useful overall they found the practical support on a scale of: “*very helpful*,” “*helpful*,” “*neither helpful or unhelpful*,” “*unhelpful*,” and “*very unhelpful*,” or could record the individual as absent. To reduce modeling complexity, the six‐level variable was condensed to either a four or three level variable: In data processing, each supporter type was labeled as either: *absent* (not applicable or provided no help); *helpful* (provided support and were rated as very helpful or helpful support); *neither* (provided support and were rated as neither helpful or unhelpful); and *unhelpful* (provided support and were rated as unhelpful or very unhelpful). Due to intermittent small numbers of individuals reporting *neither* or *unhelpful* (please see Table S3 for a breakdown), some models' standard errors were extremely large. In these cases, *neither* was combined with *unhelpful* in a three‐level categorical variable.

A measure of informational support was collected by asking participants who gave them advice or information about breastfeeding or childcare. The individuals specified were the same as for practical support, plus general practitioner (GP; family doctor), peer‐supporters (commissioned or voluntary supporters in the community who are specifically trained to provide breastfeeding support), midwifes and health visitors. In the UK, midwives provide services during pregnancy, labor, and the neonatal period. Health visitors are public health nurses who provide incommunity care and support until the child reaches the age of two. As with practical support, participants were then asked to rate how useful they found this informational support overall, and this was transformed into a four or three‐level categorical variable depending on sample sizes in each grouping.

Participants were asked how emotionally supported they felt from the various supporters (as detailed above). The response options were: “*very supported*,” “*supported*,” “*neither supported nor unsupported*,” “*unsupported*,” “*very unsupported*,” or “*not applicable*.” As with practical and informational support, this was transformed into a four or three level categorical “helpful‐unhelpful” variable depending on sample sizes in each grouping.

#### 3.1.4 Control variables

Prior to analysis, four variables were chosen as key controls for socioeconomic status given their known relationships with breastfeeding duration (Ahluwalia et al., 2005; Binns & Scott, 2002; Ingram et al., 2002). These were as follows: (a) qualification level; (b) ethnic background; (c) partnership status, and; (d) annual income. Ethnicity and partnership status were removed due to homogeneity in the sample (95% White ethnicity, 87.9% partnered). Our final sample was relatively highly educated, with 470 (81.04%) having received a graduate or postgraduate qualification. Given this distribution we reduced the levels in this variable to two; women were recorded as having a higher education (coded as 1) or not (coded as 0). For the variable *annual income* 203 (35%) reported incomes above £60,  ​000 per year. Prior to analysis this variable was reduced down to a binary variable “high earner,” coded as 0 if annual income was below £50, 000 or 1 if above it, reflecting the median point in our data (268, 46.2% reported incomes below or at £50, 000–£60, 000).

We also controlled for breastfeeding intention and background/community level breastfeeding rates which have known influences on breastfeeding duration (DiGirolamo et al., 2005; Meedya et al., 2010; Williamson et al., 2012). Participants responded to the statement “*I planned to breastfeed my baby(ies)*” either negatively or positively. This response was coded as a binary *breastfeeding intention* variable (530 or 93.6% had planned to). Another statement was “*I knew people who were breastfeeding/had breastfed their baby(ies)*.” This response was also coded as a binary variable, with 1 if they reported knowing others who breastfed. The respondents were also asked “*Were you breastfed as a baby?*,” with the options yes (1), no (2) or do not know (3). The majority of women in this sample were breastfed (409, 70.5%), with 147 (25.4%) reporting they did not know. Finally, we controlled for number of children, as multiparous mothers often report fewer breastfeeding problems (Binns & Scott, 2002; Li et al., 2008), which have less influence on breastfeeding duration (Ingram et al., 2002). The majority of women reported having one (356, 61.2%) or two children (189, 32.6%). For analysis this was transformed into a binary *parity* variable (0 = one child, 1 = more than one child).

A “base model” was created, containing only control variables which did not cause issues of multicollinearity. Of the two socioeconomic variables, only educational level was carried forward since education was highly correlated with income and 109 respondents had not filled out any financial information, reducing our sample to *n* = 470. From the four breastfeeding background controls only two went forward to the base model: *breastfeeding intention* and *parity*. *Breastfeeding intention* was the strongest breastfeeding related predictor in a control only model and was tightly correlated with being breastfed yourself and if you knew others who breastfed their children. Parity was not correlated with any other variable and was retained in the base model. Full details of this selection process can be found in the SI code.

### 3.2 Analysis

The following analytical approach reflects the plan preregistered at https://osf.io/9apyq —minor deviations were necessary in response to the data, as fully detailed in the SI, alongside the data, analysis script and result tables.

All analyses were conducted in R version 3.6.0 (Team, 2012) using the *survival* package (Therneau, 2020) for Cox regressions. The *cox.zph* function tested for violations of the proportional hazard assumption. Nonproportional hazards were corrected by fitting strata to the relevant variables: The Schoenfeld residuals were visually inspected to select the time periods at which to stratify the data. The specific time periods are detailed in the full model results.

#### 3.2.1 Hypothesis 1: Problem models

Each problem and its association with breastfeeding termination was modeled separately, and then collectively placed into a full problems model. Given modeling complexity, we sought to reduce the number of problems by combining to two sets of similar problems. Blocked ducts and mastitis followed very similar trends in the individual models (HR = 0.291, *p* < .001 and HR 0.415, *p* < .001, respectively), and in a logistic regression mastitis was a strong predictor of blocked ducts (OR = 5.224, *p* < .001). Given that blocked ducts can (but not necessarily) precede mastitis, we combined these two problems into one variable (1 = blocked ducts and/or mastitis). Likewise, nipple thrush and sore nipples were combined into a single variable (1 = thrush and/or sore nipples) due to being similar predictors of breastfeeding duration (HR = 0.493, *p* = .053 and HR = 0.480, *p* < .001, respectively) and in a logistic regression nipple thrush was a strong predictor of sore nipples (OR = 3.100, *p* = .002). In fact, only nine individuals who reported nipple thrush did not report sore nipples, indicating the similarity between these variables and the fact that nipple thrush tended to be a painful condition.

#### 3.2.2 Hypothesis 2: Interaction models with social support

To test whether receiving support moderated the association between breastfeeding problems and the hazard of breastfeeding termination, we added an interaction term to the breastfeeding problems models. Multinomial analysis demonstrated that the three different support types (informational, practical and emotional) were tightly correlated leading to issues of multicollinearity. As a result, they were modeled separately for each source of support, for each of the five different problems. Note, this means the hazard ratios of the different types of support cannot be compared across models and a large number of models were ran, reducing our ability to test hypothesis 3. We have presented models in which the effects of helpful versus unhelpful and/or absent supporters are significantly (alpha = .05) different from one another. Given the large number of models, we have focused on broader trends in the data apparent across models, reducing the risk of Type I errors by highlighting the consistency between findings.

It is important to distinguish between a significant interaction between levels of support (e.g., unhelpful and helpful) and significant main effects. In some models, the interaction between unhelpful and helpful support is significant, indicating their 95% confidence intervals do not overlap. However, while the main effect for helpful support is often significant (as more respondents reported helpful support), giving us more confidence in the effect, the same is not true of unhelpful support. Here, the 95% confidence intervals are often wide and/or spanning 1. Thus, while we are confident about the effect of helpful support, and we know the effect of unhelpful is different to the effect of helpful we do not have the same confidence of the size or direction of the main effect of unhelpful support. Nonetheless, as the research question is about the moderation effect of helpfulness, we are more interested in the difference between the slopes of “helpful” and “unhelpful,” and we highlight wider trends in the data for the main effects.

## 4 RESULTS

### 4.1 Descriptives

The mean age of the 565 respondents included in the final models was 32.28 (*SD* = 4.37) years, and they had a mean number of 1.46 (*SD* = 0.64) children. The mean age in weeks of the focal child was 50.29 (*SD* = 27.73), the youngest being 1.86 weeks and the oldest 106.29 weeks old. These women had been breastfeeding for an average of 37.04 (*SD* = 66.85) weeks, or 8.55 months. At the time of survey, 518 mothers had infants aged over 3 months, and of these 78.19% breastfed for 12.999 weeks. Of the remaining 48 mothers with infants aged less than 3 months, 93.75% were still providing breast milk.

**FIGURE 1:**
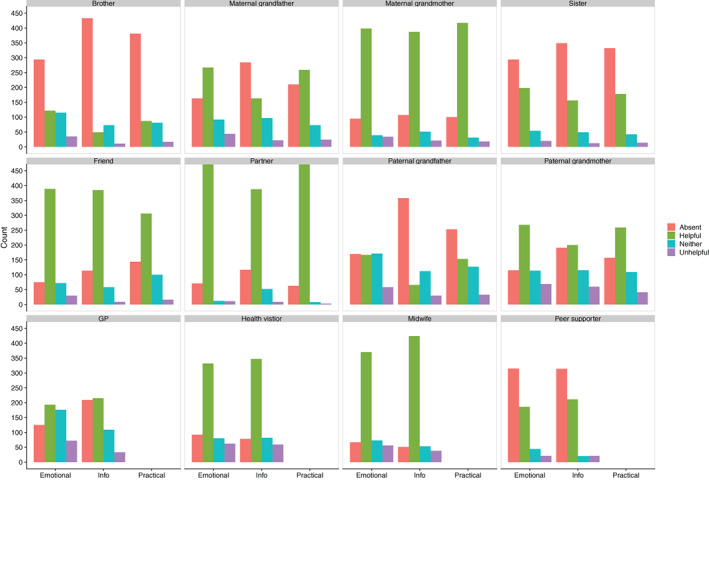
Bar chart of helpfulness of support by supporter type. Helpfulness (from bars left to right) is labeled as either absent, helpful, neither helpful or unhelpful, and unhelpful

Helpful and very helpful were the most frequently reported levels of support across supporters and domains of support (Figure 1). Partners, particularly in practical (87.28%) and emotional support (472, 83.39%) were most frequently cited as 'helpful', followed by maternal grandmothers, health visitors, and midwives. Absence of support was common for support from brothers, sisters, paternal grandmothers, and both grandfathers (own and partner's) and peer supporters. 'Unhelpful' support was the least frequent, particularly from partners, sisters, and peer supporters. Emotional support and, to a lesser degree, informational support from GPs demonstrated a different pattern; 'neither unhelpful or helpful' and 'unhelpful' were closer in frequency to that of 'helpful'. In fact, GPs had the highest frequency of 'unhelpful' levels of emotional support (72, 12.72%). Support from the paternal grandparents was also more likely to be reported as 'unhelpful', and the frequencies of 'helpful' and 'unhelpful' support were much more similar, reflecting the trend in GPs. Among family members and friends, practical (especially from the most “helpful” supporters, maternal grandmothers and partners) and emotional support had the highest frequencies of being reported as 'very helpful or helpful'.

**FIGURE 2:**
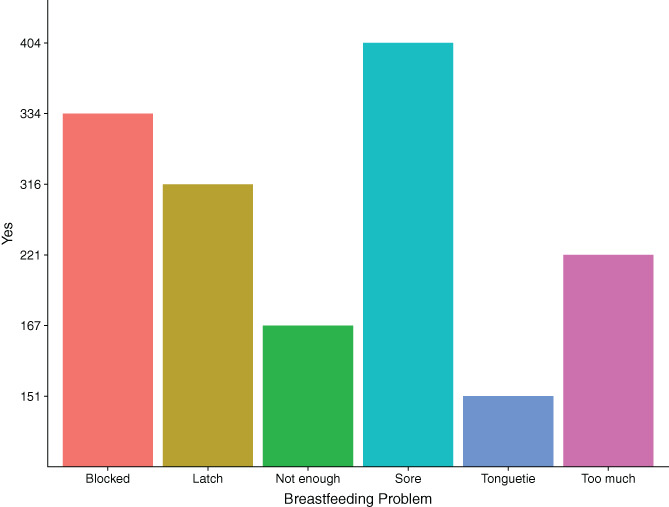
Bar chart of frequency of reported problems. 71.37% of women reported sore nipples, 40.98% reported blocked ducts, 55.83% reported latching difficulties, 39.05% reported too much breast milk, 29.51% reported not enough breast milk, and 26.67% reported their infants having tongue‐tie

Only 37 mothers (6.54%) *did not* experience any breastfeeding problems. Most women (47.88%) experienced 2 or 3 problems (mean = 2.871, *SD* = 1.624, mode = 2) and 32.69% experienced 4 to 8 separate issues when breastfeeding. The most common issue experienced was sore/cracked/blistered nipples and thrush infection in the breast (404 women, 71.38%), while the least common was tongue‐tie (151, 26.68%, Figure 2).

### 4.2 Hypothesis 1: Breastfeeding problems increase the likelihood of cessation

**FIGURE 3:**
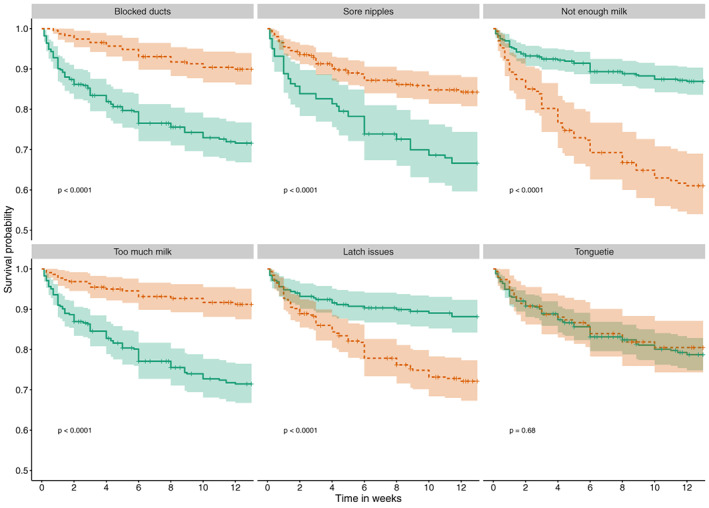
Survival curves of breastfeeding duration separated by problem type from univariate models. Solid (green) line represents when this problem was not experienced, the dashed (orange) line represents HR of cessation when problem was experienced, colored areas are 95% confidence intervals

In the full Cox regression model with all six problems (bivariate models shown in Table S2), after controlling for breastfeeding intention, maternal education and parity, the hazard of breastfeeding cessation in any given week was 3.1x higher in mothers who experienced latching problems (HR = 3.124, *p* < .001, 95% CI [2.015, 4.842], Figure 3). Likewise, mothers who reported not having enough breast milk had a cessation hazard which was 2.3x higher per week (HR = 2.303, *p* < .001, 95% CI [1.567, 4.842]). In contrast, having too much breast milk was negatively correlated with breastfeeding cessation (HR = 0.409, *p* < .001, 95% CI [0.244, 0.686]), as were blocked ducts (HR = 0.382, *p* < .001, 95% CI [0.237, 0.616]) and sore nipples (HR = 0.550, *p* = .003, 95% CI [0.370, 0.818]). Tongue‐tie was the only problem not significantly associated with cessation (HR = 0.948, *p* = .808, 95% CI [0.614, 1.463]). Based on these findings, tongue‐tie was dropped from further models.

### 4.3 Hypothesis 2: Social support will moderate the negative relationship between breastfeeding problems and cessation

For blocked ducts, helpfulness showed a consistent trend toward (though not always reaching *p* < .05) having a moderating effect on the hazard of stopping breastfeeding across both support type (Table 1) and supporters (Table 2 summarises all interaction models, visualised in  S1–S6), with only unhelpful support in conjunction with blocked ducts increasing the hazard of stopping. To take the partner model as an example, the hazard of cessation was significantly (*p* = .001) higher in mothers who experienced blocked ducts and reported unhelpful versus helpful informational support from partners; unhelpful support was associated with an increased hazard of cessation (HR = 16.826, *p* = .023, 95% CI [1.467, 193.06]), while helpful support was assoicated with a reduced hazard (HR = 0.271, *p* < .001, 95% CI [0.148, 0.494]). This is replicated in practical support from partners (*p* = .051): unhelpful HR = 8.481, *p* = .132, 95% CI [0.525, 136.9] versus helpful HR = 0.501, *p* = .020, 95% CI [0.279, 0.897].

**TABLE 1:** Cox regression interaction models set 1

Variables			Main effects	
Supporter	Support and Problem	Helpfulness	HR	*p*
Sister	Info and blocked ducts	Absent*	**0.463**	**.005**
		Unhelpful	0.35	.104
		Helpful*	**0.085**	**.001**
Friends	Practical and blocked ducts	Absent*	**0.123**	**.001**
		Unhelpful	0.418	.321
		Neither*	0.667	.376
		Helpful	**0.332**	**.001**
Friends	Info and blocked ducts	Absent*	**0.14**	**.001**
		Unhelpful*^	2.979	.286
		Neither	0.651	.435
		Helpful^	**0.307**	**<.001**
Partner	Info and blocked ducts	Absent*	**0.276**	**.019**
		Unhelpful*^§	**16.826**	**.023**
		Neither§	0.569	.297
		Helpful^	**0.271**	**<.001**
Partner	Practical and blocked ducts	Absent	0.000	.994
		Unhelpful*	8.481	.132
		Helpful*	**0.501**	**.02**
Paternal grandfather	Practical and blocked ducts	Absent	0.746	.608
		Unhelpful*	0.91	.838
		Helpful*	**0.131**	**.006**
Midwife	Info and too much milk	Absent*	**0.099**	**.028**
		Unhelpful*^	1.191	.732
		Neither	0.321	.143
		Helpful^	**0.263**	**<.001**
GP	Emotional and latch	Absent*	1.194	.802
		Unhelpful*^	**26.992**	**.001**
		Helpful^	2.205	.061
Health visitor	Emotional and too much milk	Absent*	1.196	.845
		Unhelpful^	0.582	.285
		Helpful*^	**0.056**	**.005**
Health visitor	Info and too much milk	Absent	**0.149**	**.012**
		Unhelpful*	0.704	.39
		Helpful*	**0.209**	**<.001**
Health visitor	Info and sore	Absent	0.385	.053
		Unhelpful*	1.165	.798
		Neither	0.502	.144
		Helpful*	**0.321**	**<.001**

*Note*: Presents the main effect HR and *p*‐values. Statistically significant interactions between to levels of helpfulness of indicated dyadically with *, ^, or §. When two levels share a symbol this indicates the slopes differed significantly. In the first example, this shows that the helpful information support from sisters when mothers experienced blocked ducts was associated with low risks of cessation, which is significantly different to when the support was absent. No significant relationship was found between absent and unhelpful, or helpful and unhelpful. *p*‐values which are <.05 are in bold. Where models are split into two time points only the later time results are shown as trends are consistent.

**TABLE 2:** Summary of interaction models

Supporter	Support type	Blocked ducts	Latch difficulties	Not enough milk	Sore nipples	Too much milk
Partner	Emotional					
	Informational	<				
	Practical	<				
Mother	Emotional					
	Informational					
	Practical					
Father	Emotional					
	Informational					
	Practical					
Sister	Emotional			>		
	Informational	<				
	Practical			>		
Brother	Emotional					
	Informational					
	Practical					
Friends	Emotional					
	Informational	<				
	Practical	Absent lowest				
Partners mother	Emotional					
	Informational					
	Practical			>		
Partners father	Emotional					
	Informational					
	Practical	<				
GP	Emotional		<			
	Informational					
Health visitor	Emotional			>		<
	Informational				<	<
Midwife	Emotional			>		
	Informational			>		<
Peer supporter	Emotional	Absent lowest				
	Informational	Absent lowest	Absent lowest	Absent lowest		

*Note: The light gray < indicates that helpful support decreased the hazard of stopping and dark gray > indicates helpful support increased it. The mid‐gray shade represents “absent lowest” which was when there was a significant interaction between absent support and helpful support, indicating that the lowest hazard of stopping was associated when that help was absent. Blank spaces indicate when there was no significant interaction*.

As with blocked ducts, the problems of too much breast milk and sore nipples also demonstrated the trend in which the HR for cessation was significantly reduced with helpful support as compared with unhelpful support from HCP (Table 1). For midwives, unhelpful informational support and too much breast milk had a HR of 1.191 (*p* = .732, 95% CI [0.437, 3.246]), compared with HR = 0.263 (*p* < .001, 95% CI [0.138, 0.501]) when information was reported to be helpful (interaction effect *p* = .013). While for unhelpful emotional support (HR = 0.582, 95% CI [0.215, 1.571], *p* = .285) and informational support (HR = 0.704, 95% CI [0.316, 1.569], *p* = .390) from health visitors, the confidence intervals for the main effects spanned one, the effects of unhelpful support were significantly (emotional *p* = .039 and informational *p* = .026) higher than helpful support (emotional HR = 0.056, 95% CI [0.007, 0.412], *p* = .011; informational HR = 0.209, 95% CI [0.103, 0.424], *p* < .001). Finally, health visitor's helpful informational support when a woman suffered from sore nipples meant, for every week of breastfeeding, the hazard of cessation was 67.9% lower (HR = 0.321, *p* < .001, 95% CI [0.197, 0.525]), and when this support was unhelpful the hazard was 16.5% higher (HR = 1.165, *p* = .798, 95% CI [0.362, 3.753]), which represented a significantly different slope (*p* = .047).

Another clear result in the predicted direction was emotional support from GPs (Table 1); after two and a half weeks, mothers with latch issues who reported unhelpful emotional support were significantly more likely to stop breastfeeding before 3 months (HR = 26.992, *p* = .001, 95% CI [3.656, 199.264]). This effect varies significantly (*p* = .023) from that of helpful emotional support (HR = 2.205, *p* = .061, 95% CI [0.964, 5.043]). Latch difficulties were otherwise largely unaffected by receipt of support.

The overall effect of 'not enough breast milk', which was associated with an increased likelihood of cessation, appeared amplified with support, particularly emotional support (Table 3). This is particularly evident in female family members (sisters and partner's mother) and midwifes and health visitors. The HR of breastfeeding cessation was highest for helpful support, and often significantly higher than for absent and unhelpful support. For instance, for emotional support from sisters, for each week of breastfeeding, the hazard of cessation in association with not enough milk was 6.6 times higher when support was helpful (HR = 6.558, *p* < .001, 95% CI [3.298, 13.040]), which was significantly higher than both when support was absent (HR = 2.640, *p* < .001, 95% CI [1.566, 4.452], interaction effect *p* = .039) and unhelpful (HR = 0.779, *p* = .784, 95% CI [0.161, 3.967], interaction effect *p* = .018).

**TABLE 3:** Cox regression interaction models set 2

Variables			Main effects	
Supporter	Support and Problem	Helpfulness	HR	*p*
Sister	Emotional and not enough milk	Absent^	**2.64**	**<.001**
		Unhelpful*	0.799	.784
		Neither	2.701	.086
		Helpful*^	**6.558**	**<.001**
Sister	Practical and not enough milk	Absent	**3.01**	**<.001**
		Unhelpful*	1.028	.96
		Helpful*	**5.95**	**<.001**
Paternal grandmother	Practical and not enough milk	Absent	**3.449**	**<.001**
		Unhelpful*	1.848	.089
		Helpful*	**4.984**	**<.001**
Midwife	Emotional and not enough milk	Absent	1.374	.784
		Unhelpful*	1.393	.586
		Neither^	1.106	.887
		Helpful*^	**11.884**	**<.001**
Midwife	Info and not enough milk	Absent^	**5.247**	**.009**
		Unhelpful*^	0.94	.89
		Neither	2.563	.11
		Helpful*	**4.417**	**<.001**
Health visitor	Emotional and not enough milk	Absent	**2.523**	**.047**
		Unhelpful*	1.01	.982
		Neither^	**3.66**	**.005**
		Helpful*^	**5.376**	**<.001**
Peer	Emotional and blocked ducts	Absent*^	**0.223**	**<.001**
		Unhelpful^	0.88	.792
		Helpful*	0.852	.765
Peer	Info and blocked ducts	Absent*^	**0.286**	**.011**
		Unhelpful	0.312	.271
		Helpful*	3.773	.089
Peer	Info and latch	Absent*	**3.237**	**<.001**
		Unhelpful*	0.676	.561
		Neither	0.837	.828
		Helpful	**4.795**	**.037**
Peer	Info and too much milk	Absent*	**0.252**	**<.001**
		Unhelpful*	1.219	.77
		Neither	0.704	.749
		Helpful	**0.176**	**.02**

*Note*: Presents the main effect HR and *p*‐values. Statistically significant interactions between to levels of helpfulness of indicated dyadically with *, ^, or §. When two levels share a symbol this indicates the slopes differed significantly. *p*‐values which are <.05 are in bold. Where models are split into two time points only the later time results are shown as trends are consistent.

Overall, Peer supporters followed a different trend to other supporters (Table 3). When their support was absent, for instance with informational support and blocked ducts, the hazard of cessation (HR = 0.286, *p* = .011, 95% CI [0.110, 0.747]) was significantly lower (*p* = .005) than when support was helpful (HR = 3.773, *p* = .089, 95% CI [0.815, 17.465]). Likewise, with informational support for latch difficulties and emotional support for blocked ducts.

### 4.4 Hypothesis 3: The type of support impacts the moderating effect

We could not formally test the relative roles of informational, emotional, and practical support because it was statistically inappropriate to place them in the same model as planned due to issues of multicollinearity (see SI). However, we can explore the general trends to see which type of support showed moderation effects most often. We predicted that practical and emotional support would be more important moderators than informational support, however this does not seem to be the case. The most frequent moderator (11 cases) was informational support, for which helpful support had a significantly lower HR of cessation than unhelpful support in 72% of cases. We also see that moderating informational support was provided by professionals (peer supports, health visitors, and midwives), as expected, but also from partners, sisters and friends.

## 5 DISCUSSION

In our sample of 565 relatively affluent and well‐educated women from the UK, we find that *almost everyone reported problems breastfeeding*, underlining that breastfeeding problems are the norm rather than the exception. Such problems potentially explain why, as reported elsewhere, 71% of respondents who stopped breastfeeding prior to 8 weeks found infant feeding stressful and 60% emotionally draining (Myers et al., 2021). As women often report feeling unprepared, abnormal and isolated when they encounter common issues (Brown, 2016; Wall, 2001), every attempt should be made to inform women antenatally that breastfeeding is a learnt behavior (Varki et al., 2008) and how best to prepare for, and overcome, future challenges (Brown, 2016; Emmott et al., 2020b).

The women in this study were, overall, well‐supported. Unhelpful support was rarely reported, and women frequently indicated a wide range of supporters as providing helpful support. Partners were documented as particularly helpful, as were midwives and health visitors. Beyond these well‐established supporters, we also see that the mother's mothers (infant's maternal grandmothers), friends and sisters were also providing helpful support across informational, practical and emotional domains. This indicates that multidimensional social support is an important feature of the postnatal period in the UK, even though women, especially those who are more highly educated, frequently live further from family and friends (Chan & Ermisch, 2015). As we have argued previously (Emmott et al., 2020a, 2021; Emmott & Mace, 2015; Myers et al., 2021), the benefit of an evolutionary anthropological approach is the exploration of investment transfers to mothers from a wide range of supporters due to the emphasis on cooperative childrearing (Emmott & Page, 2019). An evolutionary framework highlights that there are many mechanisms by which support can work, and support which can facilitate breastfeeding need not be limited to health care professionals.

### 5.1 Hypothesis 1: Breastfeeding problems increase the likelihood of cessation

While breastfeeding problems are frequently reported as reasons to stop breastfeeding (Dewey et al., 2003; DiGirolamo et al., 2005; Verronen, 1982), our results highlight that different types of problems have different associations with breastfeeding cessation. As is demonstrated consistently in other studies (Ahluwalia et al., 2005; Ingram et al., 2002; Kirkland & Fein, 2003; Lamontagne et al., 2008), we find that women who reported not having enough breast milk, or struggling to obtain a good latch, were much more likely to terminate breastfeeding within 3 months. These two problems can be understood as relating to infants' nutritional intake (Ingram et al., 2002; Kirkland & Fein, 2003; Li et al., 2008; Verronen, 1982), as a poor latch can result in less efficient feeding. Some have suggested that insufficient milk is often provided as a reason for stopping breastfeeding because the focus on the infant's wellbeing is a more “acceptable” reason to terminate breastfeeding within a society which often relates breastfeeding to “good” mothering (Ingram et al., 2002; Whelan & Lupton, 1998). While we have no data that can directly speak to whether insufficient milk was overreported, it is worth noting it was the second least reported problem. We also did not ask women *why* they stopped breastfeeding, thus reducing the motivation to inaccurately report and increasing confidence that insufficient milk was perceived to be an actual problem by participants. In contrast, we found that women who reported having problems with blocked ducts, mastitis, sore nipples or too much breast milk were *less* likely to terminate breastfeeding. These problems can be conceptualized as relating more to mothers' (not insignificant) pain and discomfort, or issues which require management (i.e., pumping excess milk) (Binns & Scott, 2002). Some women may be more able to endure issues of breastfeeding when it is at their own cost, rather than their infant's, which may relate to common parenting ideals focused on intensive mothering—a primarily white, middle‐class emphasis on child centered and self‐sacrificial parenting (Reyes‐Foster & Carter, 2018). Therefore, as argued by Fahy and Holschier (1988), successful breastfeeding occurs *not* in the absence of problems, but in the mother's ability to overcome these problems (Binns & Scott, 2002).

### 5.2 Hypothesis 2: Social support will moderate the negative relationship between breastfeeding problems and cessation

In the un‐moderated model, blocked ducts and too much breast milk were associated with reduced hazards of breastfeeding termination. However, by exploring the interactions between social support and breastfeeding problems, it was evident that this effect was driven by support that was considered helpful. Across the blocked ducts models, the lowest hazards of stopping breastfeeding were associated with helpful practical and informational support. Furthermore, women who had issues around blocked ducts or too much breast milk and reported unhelpful informational support (from friends, partners and midwives) were *more* likely to stop breastfeeding prior to 3 months. It has been reported elsewhere that inconsistent conflicting advice from HCPs results in mothers becoming frustrated, confused and more likely to cease breastfeeding (Garner et al., 2016; Ingram et al., 2002; Lamontagne et al., 2008). While we cannot speak to the specifics of the informational support received by our participants, our results nonetheless indicate that this effect may go beyond HCPs, with unhelpful advice from partners and friends having similar effects.

Contra to predictions, rather than reducing the negative impact of milk insufficiency on duration, support of all types—but particularly emotional support—from sisters, health visitors and midwives was associated with increased likelihoods of cessation. While the direction of causality is unknown, this relationship may be related to a woman's support needs. After experiencing perceived milk insufficiency mothers may wish to switch to formula to ensure infant's weight gain. Thus, informational and practical support may help them wean their infants. Supporters who empathize with a mother's situation or affirm her decision to stop breastfeeding may be experienced as being emotionally supportive. Levels of breastfeeding intent in our sample were high (Myers et al., 2021), potentially increasing the importance of emotional support for women who decide to stop early. This may be particularly important in a UK context where breastfeeding is currently heavily promoted, thus to cease breastfeeding is to go against a central tenant of the prevailing, White middle‐class mothering discourse (Crossley, 2009; Faircloth, 2015; Kukla, 2006; Lee, 2007). While our study did not explore women's mental wellbeing, the relationship between breastfeeding expectations, problems and postnatal depression has been well documented (Brown et al., 2016; Shakespeare et al., 2004). This highlights that social support during the postnatal period is not only about prolonging breastfeeding but also about supporting mothers mental wellbeing (Emmott et al., 2020b; Trickey, 2018; UNICEF, 2018).

As indicated above, emotional support goes beyond friends and family. In our interaction models, emotional support moderated the relationship between various problems and duration when it originated from all types of health professionals. Graffy and Taylor (2005), in a qualitative analysis of interviews with 654 women from the UK, report that although women requested informational support, women were more often sensitive to the way HCPs treated them. Alongside practical tips and guidance, they wanted acknowledgement of their experiences and to be reassured that issues during breastfeeding were normal, and thus were encouraged to continue. Clearly, emotional support from HCPs has an important role to play. This is highlighted by the negative impact that poor emotional support from GPs had in our study. Emotionally unsupportive GPs could be increasing maternal stress, making breastfeeding more difficult and further adding to mothers' concerns. Conversely, emotional support, through acknowledgement, reassurance and encouragement (Graffy & Taylor, 2005) may increase a mother's self‐efficacy to deal with latching, or other issues.

Support from a wide range of sources interacted with breastfeeding problems to predict duration. However, no significant effects were found for the mother's mother, father and brothers. This result is perhaps less surprising for brothers, given their lower levels of helpful support apparent in Figure 1, but much more surprising for mothers' mothers as they have been identified as key supporters of women in the postpartum period and beyond (Scelza, 2009; Scelza & Hinde, 2019; Sear & Coall, 2011; Sear & Mace, 2008; Snopkowski & Sear, 2015). It may be that our sample lacks the variance required to explore our question in relation to mother's mothers, as the vast majority of women reported helpful support from them. Further analyses in a more diverse sample may help unpack this. Peer supporters also demonstrated a different trend, in which the hazard of termination was the lowest when their support was absent. This probably stems from the fact that unlike GPs, health visitors and midwives, not everyone will encounter a peer supporter (as indicated in Figure 1) and likely only do so when they are facing considerable problems. Therefore, those people receiving help from peer supporters may already be more likely to stop breastfeeding.

### 5.3 Hypothesis 3: The type of support impacts the moderating effect

Practical support is framed within evolutionary approaches as reducing, or having the potential to reduce, a mother's workload, meaning mothers will have more energy to invest in tasks which maximize lifetime fitness, as construed in the current environment (Emmott & Page, 2019; Kramer & Veile, 2018; Meehan et al., 2013; Page et al., 2021). Consequently, while not true of all types of practical support (e.g., allofeeding—individuals other than the mother feeding the infant [Emmott & Mace, 2015; Myers et al., 2021]), overall, practical support is hypothesized to increase breastfeeding duration. In our sample, in which the majority expressed a wish to breastfeed (Myers et al., 2021), mothers who received helpful practical support from family members may have been able to focus on breastfeeding. This additional energy devoted to breastfeeding may have allowed them to work through problems, providing the time required to access specialist breastfeeding support.

Interestingly, in our sample, moderating practical support was received from the partner's parents (the infant's paternal grandparents), rather than the mothers' parents (the infant's maternal grandparents). Who helps mothers is likely to be partially context specific; nonetheless, evolutionary theories of kinship do predict differential investment by paternal and maternal grandparents (Beise, 2005; Gibson & Mace, 2005; Sear, 2008; Sear & Coall, 2011), suggesting that relatives from different lineages may invest in different types of supportive activities (Sear & Mace, 2008). However, it may also be the case that, as noted above, there is simply more variance in partner's family for us to pick up on these trends. It is also important to note that, by focusing on helpfulness, in this analysis we have explored all types of practical support collectively, combining allofeeding with other forms of practical support. Since allofeeding has been demonstrated to have a negative relationship with breastfeeding duration (Emmott & Mace, 2015), this may be confounding results in relation to supporters most likely to perform allofeeding—which in this sample are the partner and mother's mother (Myers et al., 2021).

Our data indicate that emotional and practical support were important moderators; however, contra predictions, so was informational support. While this hypothesis was not formally designed to separate the independent effects of emotional, practical and informational support, we did see that informational support was the most frequent moderator, and the receipt of helpful informational support was often associated with a lower likelihood of breastfeeding cessation. This underlines the fact that breastfeeding is a learnt behavior (Volk, 2009) and suggests while women benefit from practical and emotional support helping them to persist in spite of problems, informational support may curtail the duration of the problem itself. It may also be that the usefulness of informational support is dependent on its delivery alongside emotional support (Fallon et al., 2017; Fallon et al., 2019; Trickey, 2018). Future work should tease out these effects in greater depth.

Informational support was not limited to HCP but also received from partners, sisters and friends. Similar results have been found elsewhere; for example, Swedish mothers whose own mothers had discussed breastfeeding with them were more likely to breastfeed for longer, reporting greater increased confidence (Ekström et al., 2003). This demonstrates the importance of information and advice from a range of supporters. Consequently, researchers and public health specialists need to consider where else information is coming from and how to direct (helpful) information‐based interventions beyond the mother (Daniele et al., 2018; Negin et al., 2016; Wolfberg et al., 2004).

Our focus on the physiological experience of breastfeeding problems and the individual experience of social support is not to suggest that wider socioeconomic and political factors are not key predictors of breastfeeding, nor is it easy to untangle the impact of behavior, norms and structural factors (Palmquist & Doehler, 2014). There are large inequalities in infant feeding experience along structural lines in the UK and similar HIC (Victora et al., 2016), contributing to socioeconomic gradients in inflammation and infant weight (McDade & Koning, 2021). These inequalities exist due to cultural and religious norms around breastfeeding, particularly in public (Chang et al., 2021), access to social support (Grubesic & Durbin, 2020; Tomori, 2009), opportunity costs of breastfeeding (Hough et al., 2018; Tully & Ball, 2018), as well having convenient and quick‐to‐access places to breastfeed (Brown et al., 2020; Hauck et al., 2020). Furthermore, the experience of breastfeeding problems is unlikely to be evenly distributed as one study found that young, unmarried and non‐college educated US women were more likely to experience breastfeeding problems resulting in disrupted lactation (Stuebe et al., 2014). Therefore, our results may be underplaying the importance of breastfeeding problems given our sample of educated, affluent white women, who likely have privileged access to formal and informal social support. Here, we have demonstrated the importance of variation in social support in moderating the relationship between breastfeeding problems and duration, but further research with a more diverse sample is required to explore what causes variations in this support.

### 5.4 Limitations

We have already noted that our sample is a key limitation in this research. While 565 women is not an insignificant sample size, there was a lack of diversity in breastfeeding durations and support received. This is a product of the homogenous nature of our sample, which is largely educated, affluent and white—a clear limitation of this study. As a result, statistical power was likely an issue in our models increasing the likelihood of Type II errors. For this reason, while we often see nonoverlapping confidence intervals between helpful and unhelpful support, the intervals for unhelpful support are often wide and spanning one making interpretation difficult. Our sample was recruited online using convenience‐sampling, which likely biased it to more affluent women (Topolovec‐Vranic & Natarajan, 2016). This issue is not uncommon within survey‐based breastfeeding studies and should be addressed in future research. Middle‐class, more affluent women have the time, energy and desire to engage with scientific studies; more needs to be done to make this process low cost and desirable to a wider demographic. An additional concern with online‐based data collection are programs which automatically fill in surveys (e.g., bots) (Dupuis et al., 2019) and low effort respondents, both which are likely to occur when financial incentive is offered for survey completion (Buchanan & Scofield, 2018). No financial incentive was offered for the present survey, reducing our concern regarding bots—which is further diminished by the absence of suspiciously rapid completion times. Furthermore, the majority (78.6%) of respondents invested significant effort into responding to a number of optional open‐text questions (not used in this analysis, but utilized in Emmott et al. (2020a))—these would be expected to be skipped by low‐effort respondents and either skipped, answered incoherently, or repetitively by bots, which data exploration points against.

The second limitation is the potential for reporting bias due to the retrospective design of this study. We asked women with children aged up to 24 months of age about the problems they experienced and support they received *since* giving birth. Women may forget key early events in the light of later ones, and the perceived severity of a problem is likely impacted by the severity of later ones (Williamson et al., 2012). Further, given the retrospective nature of this study we have captured breastfeeding problems in a simplistic fashion. Our binary measure of yes/no hides variation in severity, duration, and number of occurrences as well as varying causes or exacerbating factors (such as maternal or infant factors). Prospective study designs are better‐suited to explore the causal relationship between support, problems and breastfeeding—all factors which fluctuate on a daily basis—and we encourage their future use.

## 6 CONCLUSION

Undoubtedly, social support from a broad range of supporters is important for successful breastfeeding: informational, emotional, and practical support all had buffering effects on the relationship between breastfeeding problems and termination. Women who suffered from pain, discomfort, or excess milk but received helpful support breastfed for longer. Breastfeeding duration also appears to be differentially contingent on informational, emotional, and practical support. This demonstrates the value of research questions which are designed from cross‐disciplinary perspectives. Both evolutionary anthropological and public health perspectives have value, specializing in practical and emotional/informational support, respectively, but so much more is gained in their integration.

Our findings suggest that it is not only problems which cause many women not to be able to fulfill their breastfeeding aims, but also the support they receive when they have these problems. Our sample was relatively well‐supported and had high breastfeeding rates, but many UK mothers face isolation in the postnatal period, lacking support across domains (Aarssen, 2005). This contrasts with the wealth of cross‐cultural anthropological evidence which highlights how embedded women are in local social networks which provide skills, advice, emotional and practical support before, during and after birth. We therefore suggest interventions and policies which aim to increase breastfeeding rates should consider the full wealth of women's social networks, in all the ways woman are supported. At the same time, we should be mindful that mothers require support beyond breastfeeding continuation. Our results suggest that emotional support may be an important factor in breastfeeding cessation, perhaps helping mothers reconcile their desire to stop against societal pressures to breastfeed. We therefore recommend that support is, above all, personalized and responsive to individual needs and breastfeeding goals.

## AUTHOR CONTRIBUTIONS

**Abigail Page:** Conceptualization; data curation; formal analysis; investigation; methodology; project administration; validation; visualization; writing‐original draft; writing‐review & editing. **Emily Emmott:** Conceptualization; data curation; formal analysis; investigation; methodology; project administration; validation; writing‐original draft; writing‐review & editing. **Sarah Myers:** Conceptualization; data curation; formal analysis; investigation; methodology; project administration; validation; visualization; writing‐original draft; writing‐review & editing.

## CONFLICT OF INTEREST

The authors declare no conflicts of interest.

## Supporting information


**Appendix** S1: Supporting Information

**Appendix** S2: Supporting Information

**Appendix** S3: Supporting Information

**Appendix** S4: Supporting Information

## Data Availability

The data and code that supports the findings of this study are freely accessed from https://osf.io/74erf/.
