# Supplementary material for: Testing the buffering hypothesis: Breastfeeding problems, cessation, and social support in the UK
Source: Am J Hum Biol. 2021 May 30;34(2):e23621. doi: 10.1002/ajhb.23621 (PMC11475338; doi:10.1002/ajhb.23621)
Supplement: Supplementary file 2 — Appendix S2: Supporting Information [file AJHB-34-e23621-s003.docx]

**SI for testing the buffering hypothesis: breastfeeding problems, cessation and social support**

**Page, A. E.^1*^, Emmott, E. H.^2^ and Myers, S.^2^**

^1^ London School of Hygiene and Tropical Medicine

^2^ University College London

* Corresponding author: abigail.page@lshtm.ac.uk

**Keywords:** infant feeding; social support; breastfeeding problems; cooperative childrearing

**SI Methods**

Deviations from the pre- registration document

The analytical approach used in this paper reflects a data analysis plan pre-registered at <https://osf.io/9apyq>. We were largely able to follow this plan, however a few differences arose, which we would like to document for the sake of full transparency.

*Difference 1: we did not explore the effect of the accumulative effect of breastfeeding problems on duration*

In the pre-registration document, we predicted that individuals reporting more breastfeeding problems will have a higher hazard of breastfeeding cessation. This entailed running models with an independent variable which was the total count of breastfeeding problems experienced by a woman. However, we soon release that this was one element too many in an already complicated (in terms of the number of models and interactions) paper. We thus decided to leave this unexplored for a future paper.

*Difference 2: we were unable to explore the relative effects of informational, practical and emotional support.*

For each interaction model we had originally planned to run the model with all three types of support in each mother. Multinomial analysis demonstrated that the three different support types (informational, practical and emotional) were tightly correlated leading to issues of multicollinearity. As a result, they were modelled separately for each source of support, for each of the five different problems. The original model set was envisioned as:

Model 1.1: Breastfeeding duration ~ problem1* partner.practical(0,1,2,3) + problem1*partner.emotional(0,1,2,3) + problem1*partner.infomational(0,1,2,3)

And ultimately this was broken down into three models:

Breastfeeding duration ~ problem1* partner.practical(0,1,2,3)

Breastfeeding duration ~ problem1* partner. emotional (0,1,2,3)

Breastfeeding duration ~ problem1* partner. infomational (0,1,2,3)

This means we were unable to test the prediction that different types of support would have different effect sizes (i.e. HR) on breastfeeding termination. Instead, we just looked at the number of times each type appeared to be an moderator across all models.

*Difference 3: Few individuals selected the response of ‘unhelpful’ support, making modelling difficult.*

Due to intermittent small numbers of individuals reporting support as neither helpful nor unhelpful, some models’ standard errors were extremely large. As a result the 95% confidence intervals spanned infinity, most frequently for the unhelpful category. In these cases, *neither* was combined with *unhelpful* in a three-level categorical variable. In some cases, even with neither and unhelpful combined the upper CI still hit infinity and this is reported as such rather than reducing the levels down further.

**Table S1:** Questions from the online survey

| **Variable** | **Survey question(s) and *response method*** |
| --- | --- |
| ***Infant feeding related measures*** | |
| **Intention to breastfeed** | I planned to breastfeed my baby(ies) – *Select if applies* |
| **Breastfeeding initiation** | Did you ever breastfeed your youngest child(ren) (including expressing)? *Yes, No, Prefer not to say* |
| **Duration of any breastfeeding** | Did you ever breastfeed your youngest child(ren) (including expressing)? *Yes, No, Prefer not to say*  Are you currently providing any breastmilk to your youngest child(ren), either exclusively or alongside formula and/or solids? *Yes, No*  Approximately, how long did you provide any breastmilk your youngest child(ren)? *Specify number and select unit (days, weeks, months)* |
| **Duration of exclusive breastfeeding** | Did you ever breastfeed your youngest child(ren) (including expressing)? *Yes, No, Prefer not to say*  Are you still exclusively breastfeeding your youngest child (i.e., no formula or other foods)? *Yes, No*  Approximately, how long did you exclusively breastfeed your youngest child(ren)? *Specify number and select unit (days, weeks, months)* |
| **Maternal subjective experience** | How would you describe your overall experience around feeding your youngest child(ren)? Please tick all that apply. *Option list included: ‘enjoyable’ and ‘rewarding’ (positive), and ‘stressful’ and ‘emotionally draining’ (negative). Response options are treated separately as they do not necessarily tap the same latent constructs, e.g., it may be possible to find infant feeding both rewarding and stressful.* |
| ***Social support measures*** | |
| **Practical support** | Thinking back to the first few weeks after giving birth to your youngest child(ren), did the people listed below do any of the following things regardless of how helpful it was? Please tick all that apply. *Option list included: ‘housework/chores around the house’ (domestic),* ‘*money for me and/or my child(ren)’ (financial),* ‘*gave me gifts and things for me and/or my child(ren)’ (material),* ‘*fed my baby(ies)’ (allofeeding),* ‘*generally looked after my baby(ies)’ (minding)*. |
| **Emotional support** | Thinking back to the first few weeks after giving birth to your youngest child(ren)…how emotionally supported did you feel by the following people? ‘*Very supported’*, ‘*supported’*, ‘*neither supported nor unsupported*, *unsupported*, *very unsupported*, *not applicable’*. |
| ***Demographic measures*** | |
| Year of birth | In what year were you born (yyyy)? *Open textbox* |
| Number of children | In total, how many children do you have? *Prefer not to say, 1, 2, 3, 4, 5, 6, 7, 8 or more* |
| Child age at the time of survey (weeks) | What date did you give birth to your youngest child(ren) (day/month/year)? |
| Highest level of educational attainment | What is your highest qualification level? *GCSEs or equivalent, AS/A-levels or equivalent, Graduate or equivalent, Postgraduate or equivalent, Other* |

**SI results**

Full output tables can be found in the SI excel sheet. In this sheet, each tab has the models for different supporters, ordered by problem type. Each model has three or four outputs associated with it; this is the same model ran three or four times (depending on if neither was combined with unhelpful, or not) with different reference values to obtain the main effects as well as the interaction effects. These are the unformatted direct outputs from R.

**Table S2:** Bivariate survival models of breastfeeding problems on breastfeeding duration

| **Problem** | **HR** | **p-value** | **L 95% CI** | **U 95% CI** |
| --- | --- | --- | --- | --- |
| Latch | 2.5162 | 1.74e-05 | 1.651588 | 3.833432 |
| Not enough | 3.430 | 5.47e-11 | 2.372978 | 4.957628 |
| Too much | 0.2724 | 2.25e-07 | 0.1664984 | 0.4456906 |
| Blocked ducts | 0.3008 | 4.87e-06 | 0.1797279 | 0.5035671 |
| Mastitis | 0.4292 | 0.00409 | 0.2409772 | 0.7645558 |
| Sore nipples | 0.4447 | 1.45e-05 | 0.3082842 | 0.6414265 |
| Nipple thrush | 0.4423 | 0.0365 | 0.2059193 | 0.9499559 |
| Tongue-tie | 0.91515 | 0.68 | 0.6007725 | 1.394044 |

**Table S3: raw frequencies of different types of support prior to data cleaning**

| **Supporter** | **Support type** | **Helpfulness** | **n** | **%** | **Supporter** | **Support type** | **Helpfulness** | **n** | **%** |
| --- | --- | --- | --- | --- | --- | --- | --- | --- | --- |
| Partner | Emotional | Absent | 71 | 12.54% | Sister | Emotional | Absent | 294 | 51.94% |
|  |  | Very helpful | 391 | 69.08% |  |  | Very helpful | 106 | 18.73% |
|  |  | Helpful | 81 | 14.31% |  |  | Helpful | 92 | 16.25% |
|  |  | Neither | 12 | 2.12% |  |  | Neither | 54 | 9.54% |
|  |  | Unhelpful | 9 | 1.59% |  |  | Unhelpful | 8 | 1.41% |
|  |  | Very unhelpful | 2 | 0.35% |  |  | Very unhelpful | 12 | 2.12% |
|  | Practical | Absent | 63 | 11.13% |  | Practical | Absent | 332 | 58.66% |
|  |  | Very helpful | 425 | 75.09% |  |  | Very helpful | 94 | 16.61% |
|  |  | Helpful | 67 | 11.84% |  |  | Helpful | 84 | 14.84% |
|  |  | Neither | 8 | 1.41% |  |  | Neither | 42 | 7.42% |
|  |  | Unhelpful | 1 | 0.18% |  |  | Unhelpful | 6 | 1.06% |
|  |  | Very unhelpful | 2 | 0.35% |  |  | Very unhelpful | 8 | 1.41% |
|  | Info | Absent | 117 | 20.67% |  | Info | Absent | 349 | 61.66% |
|  |  | Very helpful | 239 | 42.23% |  |  | Very helpful | 65 | 11.48% |
|  |  | Helpful | 149 | 26.33% |  |  | Helpful | 91 | 16.08% |
|  |  | Neither | 52 | 9.19% |  |  | Neither | 49 | 8.66% |
|  |  | Unhelpful | 7 | 1.24% |  |  | Unhelpful | 5 | 0.88% |
|  |  | Very unhelpful | 2 | 0.35% |  |  | Very unhelpful | 7 | 1.24% |
| MGM | Emotional | Absent | 95 | 16.78% | Friends | Emotional | Absent | 75 | 13.25% |
|  |  | Very helpful | 274 | 48.41% |  |  | Very helpful | 184 | 32.51% |
|  |  | Helpful | 124 | 21.91% |  |  | Helpful | 205 | 36.22% |
|  |  | Neither | 39 | 6.89% |  |  | Neither | 72 | 12.72% |
|  |  | Unhelpful | 21 | 3.71% |  |  | Unhelpful | 21 | 3.71% |
|  |  | Very unhelpful | 13 | 2.30% |  |  | Very unhelpful | 9 | 1.59% |
|  | Practical | Absent | 100 | 17.67% |  | Practical | Absent | 144 | 25.44% |
|  |  | Very helpful | 306 | 54.06% |  |  | Very helpful | 129 | 22.79% |
|  |  | Helpful | 111 | 19.61% |  |  | Helpful | 177 | 31.27% |
|  |  | Neither | 31 | 5.48% |  |  | Neither | 100 | 17.67% |
|  |  | Unhelpful | 10 | 1.77% |  |  | Unhelpful | 10 | 1.77% |
|  |  | Very unhelpful | 8 | 1.41% |  |  | Very unhelpful | 6 | 1.06% |
|  | Info | Absent | 107 | 18.90% |  | Info | Absent | 202 | 35.69% |
|  |  | Very helpful | 194 | 34.28% |  |  | Very helpful | 214 | 37.81% |
|  |  | Helpful | 193 | 34.10% |  |  | Helpful | 58 | 10.25% |
|  |  | Neither | 51 | 9.01% |  |  | Neither | 7 | 1.24% |
|  |  | Unhelpful | 14 | 2.47% |  |  | Unhelpful | 2 | 0.35% |
|  |  | Very unhelpful | 7 | 1.24% |  |  | Very unhelpful | 83 | 14.66% |
| MGF | Emotional | Absent | 163 | 28.80% | PGM | Emotional | Absent | 115 | 20.32% |
|  |  | Very helpful | 136 | 24.03% |  |  | Very helpful | 109 | 19.26% |
|  |  | Helpful | 131 | 23.14% |  |  | Helpful | 159 | 28.09% |
|  |  | Neither | 92 | 16.25% |  |  | Neither | 114 | 20.14% |
|  |  | Unhelpful | 21 | 3.71% |  |  | Unhelpful | 47 | 8.30% |
|  |  | Very unhelpful | 23 | 4.06% |  |  | Very unhelpful | 22 | 3.89% |
|  | Practical | Absent | 210 | 37.10% |  | Practical | Absent | 157 | 27.74% |
|  |  | Very helpful | 147 | 25.97% |  |  | Very helpful | 116 | 20.49% |
|  |  | Helpful | 112 | 19.79% |  |  | Helpful | 143 | 25.27% |
|  |  | Neither | 73 | 12.90% |  |  | Neither | 109 | 19.26% |
|  |  | Unhelpful | 15 | 2.65% |  |  | Unhelpful | 21 | 3.71% |
|  |  | Very unhelpful | 9 | 1.59% |  |  | Very unhelpful | 20 | 3.53% |
|  | Info | Absent | 284 | 50.18% |  | Info | Absent | 191 | 33.75% |
|  |  | Very helpful | 52 | 9.19% |  |  | Very helpful | 53 | 9.36% |
|  |  | Helpful | 111 | 19.61% |  |  | Helpful | 147 | 25.97% |
|  |  | Neither | 97 | 17.14% |  |  | Neither | 115 | 20.32% |
|  |  | Unhelpful | 11 | 1.94% |  |  | Unhelpful | 36 | 6.36% |
|  |  | Very unhelpful | 11 | 1.94% |  |  | Very unhelpful | 24 | 4.24% |
| Brother | Emotional | Absent | 294 | 51.94% | PGF | Emotional | Absent | 170 | 30.04% |
|  |  | Very helpful | 41 | 7.24% |  |  | Very helpful | 54 | 9.54% |
|  |  | Helpful | 81 | 14.31% |  |  | Helpful | 113 | 19.96% |
|  |  | Neither | 115 | 20.32% |  |  | Neither | 171 | 30.21% |
|  |  | Unhelpful | 13 | 2.30% |  |  | Unhelpful | 33 | 5.83% |
|  |  | Very unhelpful | 22 | 3.89% |  |  | Very unhelpful | 25 | 4.42% |
|  | Practical | Absent | 381 | 67.31% |  | Practical | Absent | 253 | 44.70% |
|  |  | Very helpful | 36 | 6.36% |  |  | Very helpful | 62 | 10.95% |
|  |  | Helpful | 51 | 9.01% |  |  | Helpful | 91 | 16.08% |
|  |  | Neither | 81 | 14.31% |  |  | Neither | 127 | 22.44% |
|  |  | Unhelpful | 6 | 1.06% |  |  | Unhelpful | 16 | 2.83% |
|  |  | Very unhelpful | 11 | 1.94% |  |  | Very unhelpful | 17 | 3.00% |
|  | Info | Absent | 433 | 76.50% |  | Info | Absent | 358 | 63.25% |
|  |  | Very helpful | 14 | 2.47% |  |  | Very helpful | 18 | 3.18% |
|  |  | Helpful | 35 | 6.18% |  |  | Helpful | 48 | 8.48% |
|  |  | Neither | 73 | 12.90% |  |  | Neither | 112 | 19.79% |
|  |  | Unhelpful | 2 | 0.35% |  |  | Unhelpful | 17 | 3.00% |
|  |  | Very unhelpful | 9 | 1.59% |  |  | Very unhelpful | 13 | 2.30% |
| GP | Emotional | Absent | 125 | 22.08% | Midwife | Emotional | Absent | 67 | 11.84% |
|  |  | Very helpful | 40 | 7.07% |  |  | Very helpful | 158 | 27.92% |
|  |  | Helpful | 153 | 27.03% |  |  | Helpful | 212 | 37.46% |
|  |  | Neither | 176 | 31.10% |  |  | Neither | 73 | 12.90% |
|  |  | Unhelpful | 53 | 9.36% |  |  | Unhelpful | 41 | 7.24% |
|  |  | Very unhelpful | 19 | 3.36% |  |  | Very unhelpful | 15 | 2.65% |
|  | Info | Absent | 209 | 36.93% |  | Info | Absent | 51 | 9.01% |
|  |  | Very helpful | 53 | 9.36% |  |  | Very helpful | 202 | 35.69% |
|  |  | Helpful | 162 | 28.62% |  |  | Helpful | 222 | 39.22% |
|  |  | Neither | 109 | 19.26% |  |  | Neither | 53 | 9.36% |
|  |  | Unhelpful | 19 | 3.36% |  |  | Unhelpful | 25 | 4.42% |
|  |  | Very unhelpful | 14 | 2.47% |  |  | Very unhelpful | 13 | 2.30% |
| Health visitor | Emotional | Absent | 92 | 16.25% | Peer supporter | Emotional | Absent | 315 | 55.65% |
|  |  | Very helpful | 127 | 22.44% |  |  | Very helpful | 93 | 16.43% |
|  |  | Helpful | 205 | 36.22% |  |  | Helpful | 93 | 16.43% |
|  |  | Neither | 80 | 14.13% |  |  | Neither | 44 | 7.77% |
|  |  | Unhelpful | 44 | 7.77% |  |  | Unhelpful | 9 | 1.59% |
|  |  | Very unhelpful | 18 | 3.18% |  |  | Very unhelpful | 12 | 2.12% |
|  | Info | Absent | 78 | 13.78% |  | Info | Absent | 314 | 55.48% |
|  |  | Very helpful | 141 | 24.91% |  |  | Very helpful | 126 | 22.26% |
|  |  | Helpful | 206 | 36.40% |  |  | Helpful | 85 | 15.02% |
|  |  | Neither | 82 | 14.49% |  |  | Neither | 20 | 3.53% |
|  |  | Unhelpful | 42 | 7.42% |  |  | Unhelpful | 14 | 2.47% |
|  |  | Very unhelpful | 17 | 3.00% |  |  | Very unhelpful | 7 | 1.24% |

**SI Figures:**

**Figure S1- Brother and friends models**: the relationship between breastfeeding cessation and breastfeeding problems (right hand side: blocked ducts, latch, not enough milk, soreness/pain, too much milk) moderated by the effects of emotional (green panels), information support (orange panels) and practical support (blue panels) from Brothers (top left) and Friends (top right). HRs are log transformed and represent the change in slope from ‘helpful’ support (reference category, as indicated by the line at 0 on the y-axis). Points above the line represent an increased likelihood of cessation compared to helpful support. Points below the line represent a decreased likelihood of cessation, as compared to helpful support. Error bars represent 95% confidence intervals. Some models demonstrated non-proportional hazards, these were split into early (circle) and late (triangle) time periods. Models which were not split have square point estimates. Missing points occur when the model hit estimation issues due to small sample sizes and the 95% confidence intervals spanned infinity.

**Figure S2 - Partners father and mother models:** the relationship between breastfeeding cessation and breastfeeding problems (right hand side: blocked, latch, not enough, sore, too much) moderated by the effects of emotional (green panels), information support (orange panels) and practical support (blue panels) from Paternal grandfather (top left) and paternal grandmother (top right). HRs are log transformed and represent the change in slope from ‘helpful’ support (reference category, as indicated by the line at 0 on the y-axis). Points above the line represent an increased likelihood of cessation compared to helpful support. Points below the line represent a decreased likelihood of cessation, as compared to helpful support. Error bars represent 95% confidence intervals. Some models demonstrated non-proportional hazards, these were split into early (circle) and late (triangle) time periods. Models which were not split have square point estimates. Missing points occur when the model hit estimation issues due to small sample sizes and the 95% confidence intervals spanned infinity.

**Figure S3 – Mothers’ father and mother models:** the relationship between breastfeeding cessation and breastfeeding problems (right hand side: blocked, latch, not enough, sore, too much) moderated by the effects of emotional (green panels), information support (orange panels) and practical support (blue panels) from maternal grandfather (top left) and maternal grandmother (top right). HRs are log transformed and represent the change in slope from ‘helpful’ support (reference category, as indicated by the line at 0 on the y-axis). Points above the line represent an increased likelihood of cessation compared to helpful support. Points below the line represent a decreased likelihood of cessation, as compared to helpful support. Error bars represent 95% confidence intervals. Some models demonstrated non-proportional hazards, these were split into early (circle) and late (triangle) time periods. Models which were not split have square point estimates. Missing points occur when the model hit estimation issues due to small sample sizes and the 95% confidence intervals spanned infinity.

**Figure S4 –** **Partner and sister models:** The relationship between breastfeeding cessation and breastfeeding problems (right hand side: blocked ducts, latch problems, not enough milk, sore nipples, too much milk) moderated by the effects of emotional support (green panels), information support (orange panels) and practical support (blue panels) from partners (top left) and sisters (top right). HRs are log transformed and represent the change in slope from ‘helpful’ support (reference category, indicated by the line at 0 on the y-axis). Points above the line represent an increased likelihood of cessation compared to helpful support. Points below the line represent a decreased likelihood of cessation, as compared to helpful support. Error bars represent 95% confidence intervals. Some models demonstrated non-proportional hazards, these were split into early (circle) and late (triangle) time periods. Models which were not split have square point estimates. Missing points occur when the model hit estimation issues due to small sample sizes and the 95% confidence intervals spanned infinity.

**Figure S5 - GP and Health visitor models:** The relationship between breastfeeding cessation and breastfeeding problems (right hand side: blocked ducts, latch problems, not enough milk, sore nipples, too much milk) moderated by the effects of emotional (green panels) and information support (orange panels) from GPs (top left) and Health visitors (top right). HR are log transformed and represent the change in slope from ‘helpful’ support (reference category, indicated by the line at 0 on the y-axis). Points above the line represent an increased likelihood of cessation compared to helpful support. Points below the line represent a decreased likelihood of cessation, as compared to helpful support. Error bars represent 95% confidence intervals. Some models demonstrated non-proportional hazards, these were split into early (circle) and late (triangle) time periods. Missing points occur when the model hit estimation issues due to small sample sizes and the 95% confidence intervals spanned infinity.

**Figure S6 - Midwife and peer supporter models:** The relationship between breastfeeding cessation and breastfeeding problems (right hand side: blocked ducts, latch problems, not enough milk, sore nipples, too much milk) moderated by the effects of emotional (green panels) and information support (orange panels) from midwives (top left) and peer supporters (top right). HR are log transformed and represent the change in slope from ‘helpful’ support (reference category, indicated by the line at 0 on the y-axis). Points above the line represent an increased likelihood of cessation compared to helpful support. Points below the line represent a decreased likelihood of cessation, as compared to helpful support. Error bars represent 95% confidence intervals. Some models demonstrated non-proportional hazards, these were split into early (circle) and late (triangle) time periods. Missing points occur when the model hit estimation issues due to small sample sizes and the 95% confidence intervals spanned infinity.
